# Pure- and Pseudo-Lateral-Field-Excitation Characteristics of Relaxor Ferroelectric Single Crystal PMN-PT

**DOI:** 10.3390/mi14061136

**Published:** 2023-05-28

**Authors:** Fei Sun, Tingfeng Ma, Pengfei Kang, Yuming Yao, Ning Gan, Lili Yuan, Wenhui Hu, Iren Kuznetsova, Ilya Nedospasov

**Affiliations:** 1 School of Mechanical Engineering and Mechanics, Ningbo University, Ningbo 315211, Chinakangpengfei22@163.com (P.K.);; 2 Keli Sensing Technology (Ningbo) Co., Ltd., Ningbo 315033, China; 3 School of Material Science and Chemical Engineering, Ningbo University, Ningbo 315211, China; 4 Kotelnikov Institute of Radio Engineering and Electronics of RAS, Moscow 125009, Russia

**Keywords:** lateral-field-excitation, relaxor ferroelectric single crystal, pure-LFE, pseudo-LFE, cuts

## Abstract

The relaxor ferroelectric single crystal (1−x)Pb(Mg_1/3_Nb_2/3_)O_3_-xPbTiO_3_ (PMN-PT) has high piezoelectric constants, and thus has a good application prospect in the field of highly sensitive piezoelectric sensors. In this paper, for relaxor ferroelectric single crystal PMN-PT, the bulk acoustic wave characteristics on pure- and pseudo-lateral-field-excitation (pure- and pseudo-LFE) modes are investigated. LFE piezoelectric coupling coefficients and acoustic wave phase velocities for PMN-PT crystals in different cuts and electric field directions are calculated. On this basis, the optimal cuts of pure-LFE and pseudo-LFE modes of relaxor ferroelectric single crystal PMN-PT are obtained, namely, (*zxt*)45° and (*zxtl*)90°/90°, respectively. Finally, finite element simulations are carried out to verify the cuts of pure-LFE and pseudo-LFE modes. The simulation results show that the PMN-PT acoustic wave devices in pure-LFE mode have good energy-trapping effects. For PMN-PT acoustic wave devices in pseudo-LFE mode, when the device is in air, no obvious energy-trapping emerges; when the water (as a virtual electrode) is added to the surface of the crystal plate, an obvious resonance peak and the energy-trapping effect appears. Therefore, the PMN-PT pure-LFE device is suitable for gas-phase detections. While the PMN-PT pseudo-LFE device is suitable for liquid-phase detections. The above results verify the correctness of the cuts of the two modes. The research results provide an important basis for the development of highly sensitive LFE piezoelectric sensors based on relaxor ferroelectric single crystal PMN-PT.

## 1. Introduction

Sensors based on piezoelectric bulk acoustic resonators have been widely used in gas and liquid-phase sensing in recent years [1,2,3,4]. Traditional piezoelectric resonators usually are based on thickness-field-excitation (TFE) mode (the electrodes are located on the upper and lower surfaces of the resonator) [5,6,7,8]. In recent years, the lateral-field-excitation (LFE) mode (electrodes are distributed on the same surface of the piezoelectric plate) has been developed [9,10,11]. Devices based on the LFE mode have obvious advantages compared with those based on the TFE mode [6], such as a higher quality factor, better frequency stability, lower crystal aging rate and higher electrical sensitivity.

Previous LFE piezoelectric bulk acoustic wave sensors are mostly based on quartz crystals; however, the piezoelectric coupling coefficients of quartz crystal devices are low [12,13], resulting in limited sensitivities. Relaxor ferroelectric single crystals are increasingly important in the field of piezoelectric devices due to their high piezoelectric constants [14,15,16,17,18,19] and are considered to be a good choice of piezoelectric material for next-generation high-performance transducers and sensors. In addition, the shear mode of the PMN-PT single crystal is of practical importance in shear horizontal (SH) wave generation and reception [20], thus the PMN-PT single crystal also has a good application prospect on guided wave generation and reception. Artificial polarization is necessary so that the spontaneous polarization direction of the ferroelectrics is arranged in the direction closest to the electric field through the reorientation of the electric domain, thereby exhibiting piezoelectricity [21]. For artificial polarization, the single crystal is placed in a silicone oil bath for polarization, and the silicone oil is heated to 10°. Then it is kept warm, the DC voltage polarization is increased about three times the coercive field of the crystal (PMN-PT is about 1 kV/mm) for 15 min, and then the polarization voltage is halved and naturally cools to room temperature.

Wang et al. found that piezoelectric devices based on lateral-field-excitation have three different operating modes [22,23,24], namely, pure-LFE mode, quasi-LFE mode and pseudo-LFE mode, which were further verified by experiments. Among them, a single vibration mode can be obtained for pure-LFE mode and pseudo-LFE mode, which are suitable to be used as the operational mode of bulk acoustic wave sensors. According to Wang’s theory [22], for pure-LFE mode devices, the thickness-shear mode wave is excited only by a lateral electric field. For pseudo-LFE mode devices, when in air, there is no resonance peak; when in liquids, the resonance peak is generated not by the LFE but by the TFE with the liquid acting as a virtual electrode. Relaxor ferroelectric single crystals PMN-PT has high piezoelectric constants, which is important for obtaining high sensitivities of LFE sensors [23]. However, the LFE characteristics of relaxor ferroelectric single crystals are still unknown, and for relaxor ferroelectric single crystals, the cuts of the pure-LFE and pseudo-LFE mode are not clear, which hinders applications of relaxor ferroelectric single crystals in LFE piezoelectric sensors.

In this paper, the bulk acoustic wave characteristics of pure-LFE and pseudo-LFE devices based on relaxor ferroelectric single crystal PMN-PT with high piezoelectric coupling coefficients are calculated. The acoustic wave phase velocities and piezoelectric coupling coefficients for different cuts and electric field directions are achieved, based on which, the optimal cuts of pure-LFE and pseudo-LFE operating modes are obtained. Finally, finite element simulations are carried out to verify the cuts of pure-LFE and pseudo-LFE operating modes for PMN-PT crystals.

## 2. Lateral-Field-Excitation Characteristics of Relaxor Ferroelectric Single Crystal PMN-PT

The piezoelectric coupling coefficient reflects the efficiency of energy conversion between electrical and mechanical energies [25]. The LFE coupling factors of the PMN-PT crystals in different cuts and electric field directions have been calculated by using the extended Christoffel–Bechmann method [26]. The material constants of PMN-PT crystals are from [27].

**Figure 1:**
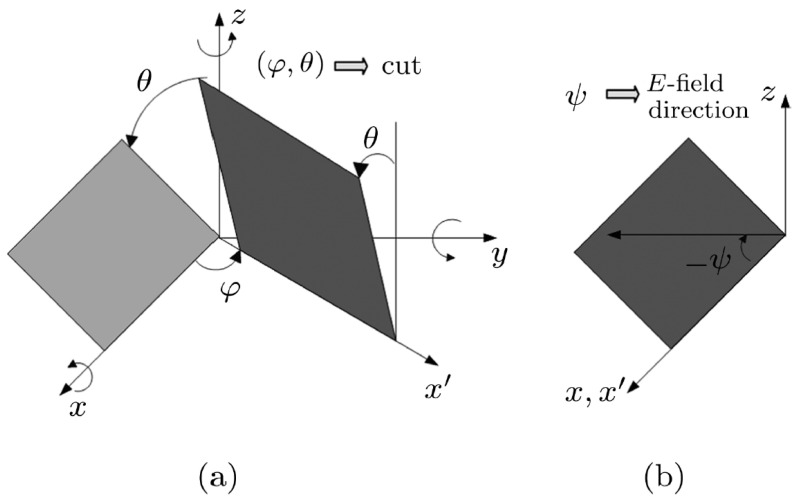
Notation system used for calculating: (**a**) crystallographic orientation and (**b**) electric field direction.

The crystal axial set and the notation (*yxwl*)φ/θ are defined according to the IEEE standard [28,29]. The orientation of a substrate aligned with a rotated coordinate set of axes is shown in Figure 1a, and the electric field direction relative to crystallographic axes is shown in Figure 1b. The IEEE standard is used to define the crystal axial set and the notation (*yxwl*)φ/θ, as shown in Figure 1.

**Figure 2:**
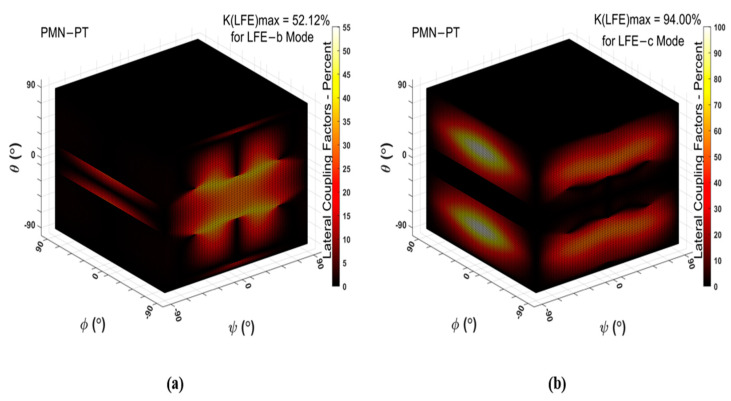
(**a**) Piezoelectric coupling coefficients of LFE-b mode of PMN-PT crystals; (**b**) piezoelectric coupling coefficients of LFE-c mode of PMN-PT crystals.

**Figure 3:**
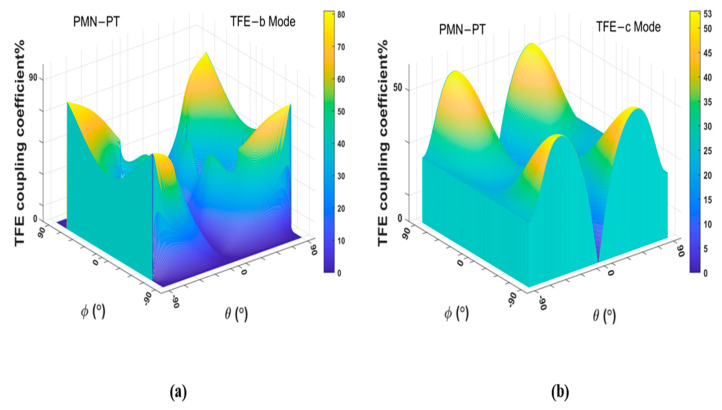
(**a**) Piezoelectric coupling coefficients of TFE-b mode of PMN-PT crystals; (**b**) piezoelectric coupling coefficients of TFE-c mode of PMN-PT crystals.

The effective vibration mode of pure-LFE mode is LFE quasi-fast shear mode (LFE-b mode) or LFE quasi-slow shear mode (LFE-c mode), and the effective vibration mode of pseudo-LFE mode is TFE quasi-fast shear mode (TFE-b mode) or TFE quasi-slow shear mode (TFE-c mode). The LFE and TFE coupling coefficients of PMN-PT crystals in all cuts and electric field directions are calculated in order to obtain the cuts of pure-LFE and pseudo-LFE modes, and the calculated results are shown in Figure 2 and Figure 3.

**Figure 4:**
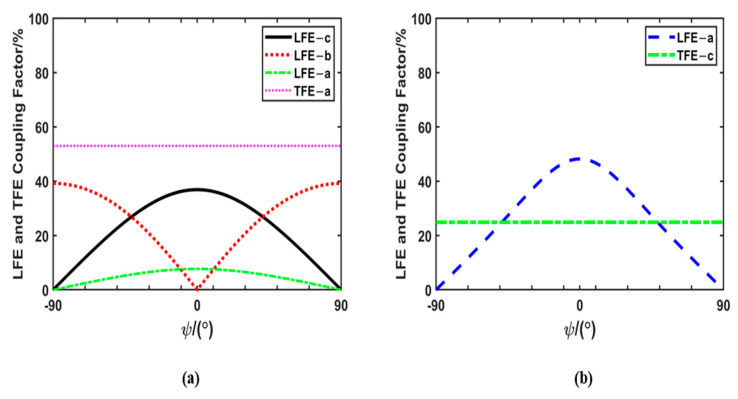
(**a**) LFE and TFE piezoelectric coupling factors of (*zxt*)45° PMN-PT as a function of the electric field angle (the coupling coefficients of TFE-b and TFE-c are approximately 0); (**b**) LFE and TFE piezoelectric coupling factors of (*zxtl*)90°/90° relaxor ferroelectric crystals as a function of electric field angle (the coupling coefficients of LFE-b, LFE-c, TFE-a and TFE-b are approximate 0).

As shown in Figure 2a, the maximum LFE coupling coefficient of mode b is 52.12%, and its corresponding cut is (*zxtlw*)±90°/±17°/0°. As can be seen from Figure 2b, the maximum LFE coupling coefficient of mode c is 94% and its corresponding cut is (*zxtlw*)0°/±45°/±90°. The piezoelectric coupling coefficient of the LFE-b mode under this cut is zero, and, as can be seen from Figure 3b, the piezoelectric coupling coefficient of the TFE-c mode of this cut is not zero. Therefore, not a single shear vibration mode can be obtained for this cut. According to the calculation, a single shear vibration mode can be obtained for the cut of (*zxt*)45°. For this cut, the variations of the LFE and TFE piezoelectric coupling coefficients with the electric field angles are shown in Figure 4a. When the electric field angle ψ = 0°, the LFE coupling coefficient of mode b is 0, and that of mode c is 36.9%. In addition, the coupling coefficients of TFE-b and TFE-c modes are approximately 0. Therefore, a single LFE shear vibration mode can be achieved for the cut of (*zxt*)45°, that is, (*zxt*)45° PMN-PT can meet the conditions of pure-LFE mode.

As shown in Figure 3a, the maximum TFE coupling coefficient of mode b is 80.9% and its corresponding cut is (*zxtl*)±72°/±90°. It can be seen from Figure 3b that the maximum TFE coupling coefficient of mode c is 53.4% and its corresponding tangent is (*zxtl*)±90°/±50°. Therefore, a larger coupling coefficient of TFE shear mode can be obtained for the cut of (*zxtl*)±72°/±90°. However, it can be seen from Figure 3b that the piezoelectric coupling coefficient of the TFE-c mode corresponding to this cut is not zero. The calculated results in Figure 4 show that for the cut of (*zxtl*)90°/90°, when the electric field angle ψ = ±90°, the piezoelectric coupling coefficient of LFE c mode and b mode is zero. The TFE coupling coefficient of mode b is 0 and that of mode c is 24.7%. A single TFE vibration mode can be obtained for (*zxtl*)90°/90°. Thus, PMN-PT LFE devices on this cut operate in pseudo-LFE mode.

## 3. Calculation of Phase Velocity of Acoustic Waves of Pure-LFE and Pseudo-LFE Mode

For PMN-PT crystals, a piezoelectrically stiffened Christoffel matrix is calculated according to Equation (1) [30].

(1) $$\Gamma_{ij}=l_{iK}\left(c_{KL}^{E}+\frac{\left(e_{Kj}l_{j}\right)\left(l_{i}e_{iL}\right)}{l_{i}\varepsilon_{ij}^{S}l_{j}}\right)l_{Lj},$$

where, *l_iK_* is the wave propagating direction matrix, $C_{KL}^{E}$ is the stiffness constants matrix under the condition of a constant electric field, $l_{j}$ is the field direction matrix, $l_{i}$ is the transpose of $l_{j}$, $e_{Kj}$ is the piezoelectric stress constants matrix, $e_{iL}$ is the transpose of $e_{Kj}$ and $\varepsilon_{ij}^{S}$ is the permittivity constants matrix under the condition of a constant strain. Then, the characteristic Equation (2) is solved.

(2) $$\left|\Gamma_{ij}-c\delta_{ij}\right|=0$$

After the three characteristic values are obtained, the characteristic values $c_{m}$ and material density ρ are substituted into Equation (3) to obtain the acoustic wave phase velocities $v_{m}$ corresponding to the modes of a, b and c, respectively.

(3) $$v_{m}=\sqrt{\frac{c_{m}}{\rho }}$$

**Figure 5:**
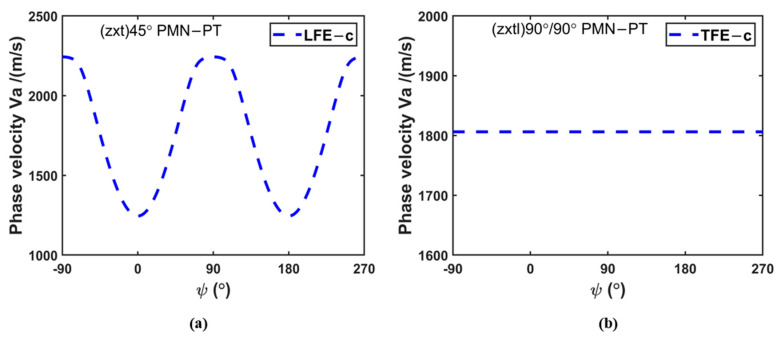
(**a**) Relationship between the acoustic wave phase velocity of the pure-LFE mode of PMN-PT crystals and the electric field angle; (**b**) relationship between the acoustic phase velocity of the pseudo-LFE mode of PMN-PT crystals and the electric field angle.

The acoustic wave phase velocities for pure-LFE mode cut (*zxt*)45° and pseudo-LFE mode cut (*zxtl*)90°/90°are obtained, which are shown in Figure 5.

As shown in Figure 5a, the acoustic phase velocity of pure-LFE mode changes sinusoidally with the change of the electric field angle. When the electric field angle is ψ = ±90° or ψ = 270°, the maximum acoustic phase velocity can be obtained, namely, 2242.9 m/s. When the electric field direction is ψ = 0°or ψ = 180°, the minimum phase velocity of acoustic wave is achieved, namely, 1245.4 m/s.

As shown in Figure 5b, the phase velocity of the pseudo-LFE mode does not change with the variation of the electric field angle, keeping 1806.3 m/s. The reason for this phenomenon is that the LFE piezoelectric coupling coefficient of the device operating on pseudo-LFE mode is zero, and the TFE coupling piezoelectric coefficient is not zero. The thickness electric field plays a major role; thus, the acoustic phase velocity does not change with the lateral electric field angle.

## 4. Energy-Trapping Effects of PMN-PT Acoustic Wave Devices on Pure- and Pseudo-LFE Modes

To verify the energy-trapping effects of PMN-PT acoustic wave devices on pure- and pseudo-LFE modes, finite element simulations using COMSOL Multiphysics (Burlington, MA, USA), a commercially available modeling package, are performed. This model is a three-dimensional model, and the model size parameters are the same as the theoretical model parameters. The frequency domain analysis is conducted to obtain the characteristic frequency of the resonator. The linearized Navier–Stokes, frequency domain interface, laminar flow interface and piezoelectric coupling field interface in COMSOL Multiphysics are employed. FSI (fluid–structure interaction) is used to model the interactions between the liquid and the resonator.

### 4.1. Pure-LFE Mode

**Figure 6:**
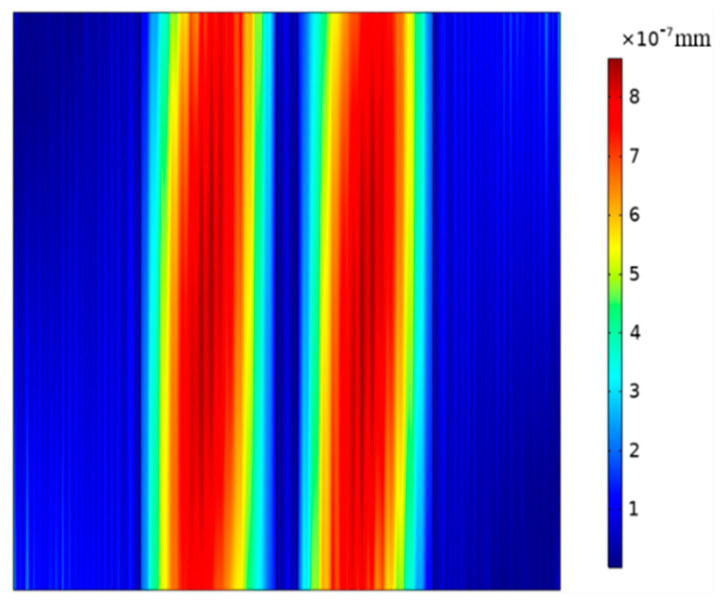
Vibration cloud image of the device operating on pure-LFE mode.

By solving the characteristic frequency by using COMSOL Multiphysics, the vibration cloud image of the device based on (*zxt*)45° PMN-PT with a resonance frequency of 5.02 MHz is obtained, as shown in Figure 6.

It can be seen from Figure 6 that the vibration energy of the resonator is mainly concentrated in the electrode regions, and the vibrations of the electrode regions do not change along the width direction, and there is almost no vibration in the non-electrode regions, indicating that the resonator has good energy-trapping characteristics.

**Figure 7:**
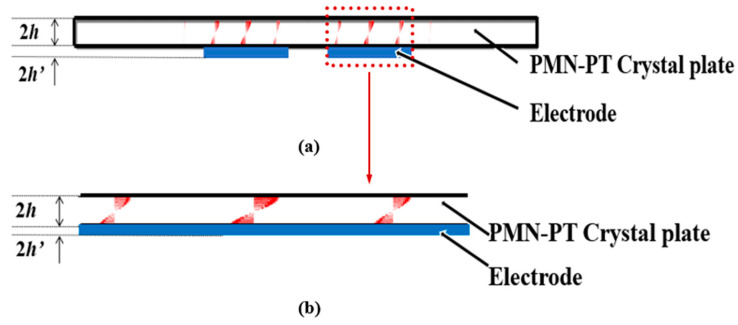
Displacement vector diagrams along the length of PMN-PT devices on pure-LFE mode: (**a**) displacement vector diagrams of the length slice; (**b**) a partially enlarged view of the length slice. (Red color represents the direction and magnitude of the particle displacement, blue color represents the electrode, and the red arrow represents that Figure 7b is the partial enlargement of Figure 7a.

**Figure 8:**
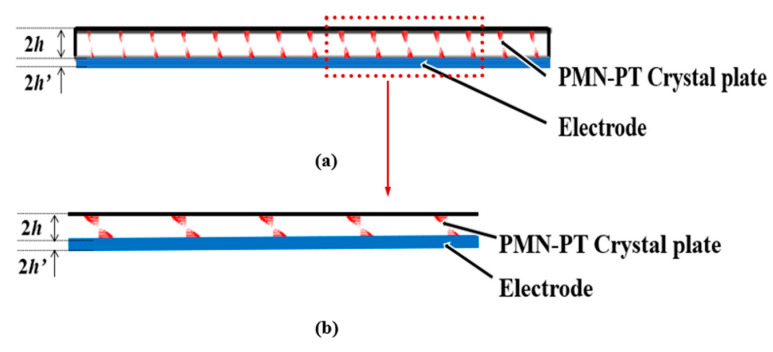
Displacement vector diagrams along the width of PMN-PT devices on pure-LFE mode: (**a**) displacement vector diagrams of the width slice; (**b**) a partially enlarged view of the width slice. (Red color represents the direction and magnitude of the particle displacement, blue color represents the electrode, and the red arrow represents that Figure 8b is the partial enlargement of Figure 8a.

Figure 7 and Figure 8 show displacement vector diagrams along the length and width of the substrate plate, respectively. The arrows are the directions of displacement. It can be seen from Figure 7a,b that the vibration is mainly concentrated in the central electrode region, and the vibration in the external non-electrode region is almost negligible. Thus, the device has a good energy-trapping effect. Moreover, there is a zero node in the thickness direction of the displacement vector diagram, indicating that there is no vibration in the central face of the substrate of the device. In addition, it can be seen from Figure 8a,b that the main vibration mode is a first-order mode, and there is no attenuation trend along the width direction. For this frequency, the main vibration mode of the device is the thickness-shear mode.

By carrying out frequency domain calculations using COMSOL Multiphysics, the admittance diagram of the device operating on pure-LFE mode is obtained, through which the precise resonant frequency of the resonator can be achieved. The admittance is defined as $Y_{m}=j\omega C_{0}K^{2}\cot\left(\frac{kd}{2}\right)/\frac{kd}{2}$, where *K* is electromechanical coupling coefficient,$C_{0}$ is static capacitance, *d* is thickness of the piezoelectric plate and *k* is the propagation coefficient.

**Figure 9:**
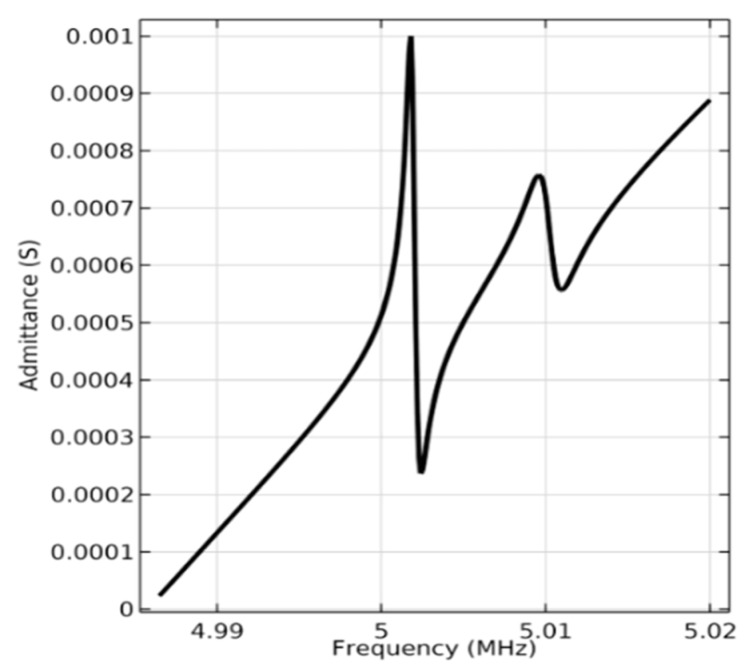
Admittance diagram of the device operating on pure-LFE mode from COMSOL.

As shown in Figure 9, the peak resonance in the admittance diagram of the resonator is 5.0018 MHz, which is close to the calculated value of 5.02 MHz obtained by the characteristic frequency calculation.

### 4.2. Pseudo-LFE Mode

**Figure 10:**
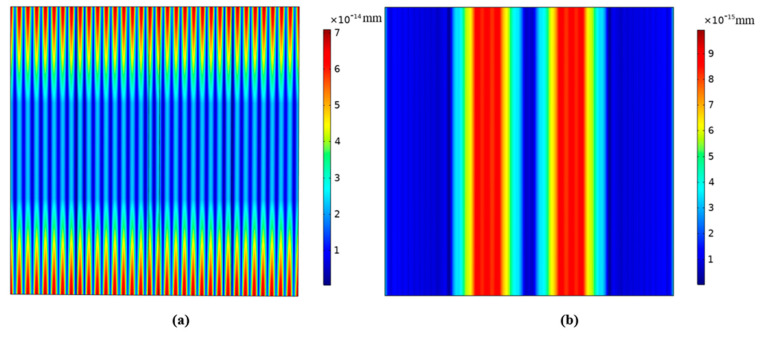
Mode diagrams of device operating on pseudo-LFE mode: (**a**) in the air; (**b**) with liquid load(water) on one side.

The vibration distribution of the (*zxtl*)90°/90° PMN-PT device in the air is obtained through calculating the characteristic frequency, as shown in Figure 10a. It can be seen from the figure that there is no energy-trapping effect near the fundamental frequency; thus, the device does not exist thickness-shear vibration mode in air.

Figure 10b shows the (*zxtl*)90°/90° PMN-PT LFE device with liquid load (water) on one side, and the resonant frequency is 4.83 MHz. It can be seen from the figure that the vibration energy of the resonator is mainly concentrated in the electrode region, and the vibration of the electrode region does not change along the width direction, and there is almost no vibration in the non-electrode region, indicating that the resonator with liquid load on one side has good energy-trapping characteristics.

**Figure 11:**
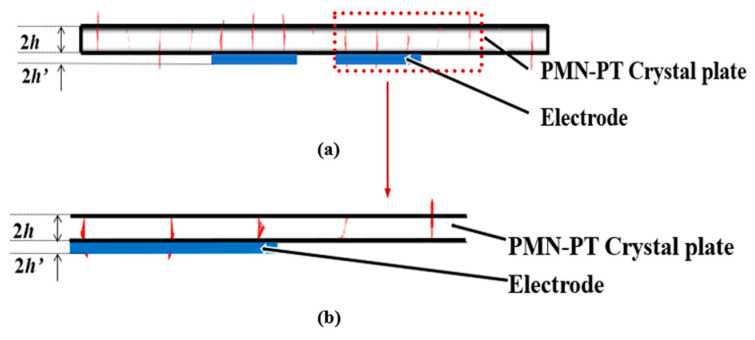
Displacement vector diagrams along the length of PMN-PT devices on pseudo-LFE mode in air: (**a**) displacement vector diagrams on the length slice of the device in the air; (**b**) a partially enlarged view of the length slice of the device in the air. (Red represents the direction and magnitude of the particle displacement, blue represents the electrode, and arrows represent the partial enlargement of Figure (b) in Figure (a)).

**Figure 12:**
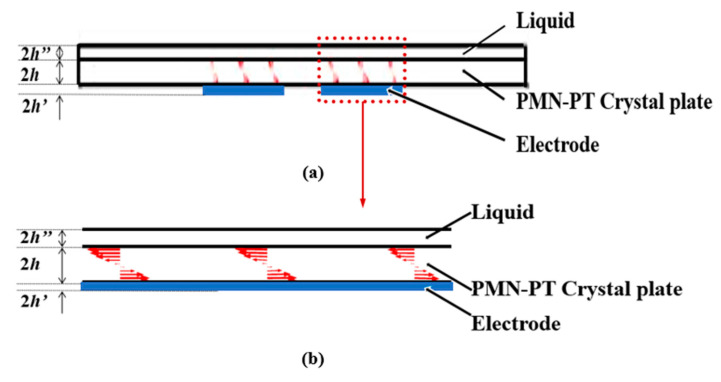
Displacement vector diagrams along the length of PMN-PT devices with liquid load (water) on one side: (**a**) displacement vector diagrams on length slice of the device with liquid load; (**b**) a partially enlarged view of the length slice of the device with the liquid load. (Red represents the direction and magnitude of the particle displacement, blue represents the electrode, and arrows represent the partial enlargement of Figure (b) in Figure (a)).

Figure 11 and Figure 12 represent vector diagrams of LFE devices based on PMN-PT crystals operating in pseudo-LFE mode in the air and with water on one side, respectively. The arrows represent the displacement vectors. It can be seen from Figure 11a,b that there is no energy-trapping effect in the air. In Figure 12a,b, it can be seen that when there is a liquid load, the vibration of the device is mainly concentrated in the central electrode region, and the vibration in the external non-electrode region is almost negligible. It can be seen that the device has a good energy-trapping effect when it is under the liquid-phase load.

## 5. Conclusions

In this paper, the bulk acoustic wave characteristics of pure-LFE and pseudo-LFE devices based on relaxor ferroelectric crystal PMN-PT are calculated, and LFE piezoelectric coupling coefficients and acoustic wave phase velocities are obtained, based on which, the cuts of the two modes are achieved. The results show that (*zxt*)45° and (*zxtl*)90°/90° are the cuts for pure and pseudo-LFE modes of PMN-PT crystals, respectively. Then the simulation results show that the pure-LFE device based on the relaxor ferroelectric single crystal has a good energy-trapping effect. For pseudo-LFE devices, no resonance peak and energy-trapping effect appear when the device is in the air. When liquid (as a virtual electrode) is added to the surface of the device, an obvious resonance peak and energy-trapping effect appear. The above results verify the correctness of the cuts of two modes of PMN-PT crystals. In addition, it is shown that the pure-LFE device can obtain good resonance characteristics when in the air, thus it is suitable for gas-phase detections. On the other hand, the pseudo-LFE device can achieve good resonance characteristics when with the liquid load, thus it is suitable for liquid-phase detections. The results can provide an important basis for bulk acoustic wave sensors operating on pure-LFE and pseudo-LFE modes based on relaxor ferroelectric single crystals.

## Data Availability

Not applicable.
